# A Feasibility and Efficacy Randomized Controlled Trial of Two Exercise Programs in Severe AECOPD Patients with Resting Hypoxemia

**DOI:** 10.3390/healthcare9091102

**Published:** 2021-08-25

**Authors:** Laura López-López, Andrés Calvache-Mateo, Janet Rodríguez-Torres, María Granados-Santiago, Araceli Ortiz-Rubio, Marie Carmen Valenza

**Affiliations:** Department of Physical Therapy, Faculty of Health Sciences, University of Granada, 18016 Granada, Spain; lauralopez@ugr.es (L.L.-L.); andrescalvache@ugr.es (A.C.-M.); jeanette92@hotmail.com (J.R.-T.); mariagranados@ugr.es (M.G.-S.); aortiz@ugr.es (A.O.-R.)

**Keywords:** COPD, chronic obstructive pulmonary disease, exacerbation, exercise training, hypoxemia, NEMS

## Abstract

Resting hypoxemia is the most severe stage of Chronic Obstructive Pulmonary Disease (COPD). Due to their impairments during the exacerbation, these patients are limited to traditional exercise rehabilitation and are excluded from the majority of the studies. The aim of this study was to assess the feasibility and the efficacy of two exercise programs in Acute Exacerbation of COPD (AECOPD) patients with resting hypoxemia. In this randomized clinical trial, patients hospitalized due to an acute exacerbation of COPD with hypoxemia at rest were included. Patients were randomly assigned into three groups. A Control Group (pharmacological treatment), a Global Exercise Group (GEG), and a Functional Electrostimulation Group (FEG). Patients were treated during the hospitalization period. The main outcomes were lower limb strength (assessed by a dynamometer), balance (assessed by the one leg standing balance test), health related quality of life (assessed by the EQ-5D), adverse events and adherence. At the end of the intervention, there were significant differences in all the variables in favour of the experimental groups (*p* < 0.05). We concluded that conducting an exercise program is feasible and improves lower limb strength, balance, and health related quality of life in AECOPD patients with resting hypoxemia.

## 1. Introduction

The prevalence of hypoxia in patients with Chronic Obstructive Pulmonary Disease (EPOC) increase as the disease worsens [1], due to the progressive airflow limitation and emphysematous destruction of the pulmonary capillary bed [2]. Different types of hypoxemia have been described in COPD patients, resting hypoxemia being the most severe stage of the disease [3].

Hypoxia is considered an important factor in many of the comorbidities that characterize COPD, such as myocardial stress due to the increased heart rate, musculoskeletal dysfunction, and systemic inflammation [4]. These comorbidities are worsened during an exacerbation [5] due to prolonged bed rest and treatment with steroids [6], showing a reduction in the quality of life, exercise tolerance, and a greater risk of death in these patients [7].

Despite this evidence, some aspects of the management in patients with hypoxemic Acute Exacerbated COPD (AECOPD) are subject to debate, such as the role of supplemental oxygen therapy [4,8] and exercise rehabilitation. Although individual exercise training is an integral component of respiratory rehabilitation [9], there is controversy over the safety and possible adverse effects in these patients.

While Rabinovich et al. [10] demonstrated that moderate exercise results in an enhanced inflammatory response in skeletal muscle, Casanova et al. [11] affirmed that desaturation with exercise appears to predict increased risk of mortality. This, along with ventilator constraints and the locomotor muscle weakness limit the participation in traditional exercise rehabilitation and are the reasons why these patients are excluded from the majority of the studies that carry out early interventions in patients with AECOPD [12,13].

Therefore, a better understanding of the safety and the efficacy of exercise training for AECOPD patients with resting hypoxemia is needed. We hypothesize that exercise training is safe and could counter the deterioration occurring during an exacerbation. Therefore, the aim of this study was to assess the feasibility and the efficacy of two exercise programs in AECOPD patients with resting hypoxemia.

## 2. Materials and Methods

### 2.1. Study Design

In this randomized single-blind clinical trial, all patients were informed of the study purposes and signed an informed consent. The research assistant who collected the data was blinded to the hypothesis of the study and to the patient’s allocation. The study was approved by the Biomedical Research Ethics Committee of Granada (code number: 00256) and in accordance with the Declaration of Helsinki. Additionally, it was registered in ClinicalTrials.gov Identifier: NCT04295655. The CONSORT guideline [14] was followed during the course of the research.

### 2.2. Randomization Procedure

Patients were randomly assigned to one of the three groups (control, global exercise, and functional electrostimulation groups). One investigator who was not involved in the study carried out the computer-generated randomization sequence. The allocation of the patients was concealed in sequentially numbered, sealed, and opaque envelopes.

### 2.3. Patients

We recruited all patients from the Respiratory Services of two hospitals for four months (March 2019 to August 2020). The inclusion criteria were: (1) Patients with a diagnosis of COPD made according to the criteria of the American Thoracic Society (ATS) [15], (2) with a moderate to severe COPD (GOLD III-IV (FEV1% < 50)), (3) hospitalized AECOPD patients [16], (4) with hypoxemia at rest defined as resting SpO2 between 89–93% [17], (5) and who agreed to participate. Exclusion criteria included severe comorbidities that could interfere with the evaluation or with the treatment, as well as contraindications of electrotherapy [18].

### 2.4. Evaluation

Demographic and clinical information about age and body mass index (BMI) was acquired from electronic patient files, interviews, and clinical assessments. Additionally, spirometry was performed on all the subjects following the criteria of the American Thoracic Society [19]. Other variables assessed were Saint George Respiratory Questionnaire (SGRQ) [20] to evaluate the quality of life, Charlson Comorbidity Index [21] to evaluate the comorbidities, and the Hospital Anxiety and Depression Scale (HAD) [22] to assess anxiety and depression.

The main outcomes measured were lower limb strength, balance, health related quality of life, adverse events and adherence, and were evaluated at admission and at discharge.

#### 2.4.1. Lower Limb Strength

Quadriceps strength was assessed with a portable hand-held dynamometer (Lafayette Manual Muscle Testing System, model 01163, Lafayette, IN, USA). The test was performed three times with each leg alternately to allow the participants to rest between measurements. The participant carried out a 5 s maximal muscle contraction in a seated position. The highest value in Newtons was selected for the analysis [23,24].

#### 2.4.2. Balance

The One-Leg Standing balance test (OLS) was used to evaluate the balance. It measured the time that the patient was able to balance on one leg. The patient chose a leg to stand on (whichever he/she felt more comfortable with), flexed the opposite knee allowing the foot to clear the floor, and balanced on one leg [25].

### 2.5. Health Related Quality of Life

Health related quality of life was assessed by the EuroQol-5D (EQ-5D). It contains two sections, a descriptive section and a valuation section. The descriptive section is composed of five dimensions with three levels of functioning: Mobility; self-care; usual activities; pain/discomfort; and anxiety/depression. The valuation section consists of a visual analogue scale ranging from 0 (defined as the worst imaginable health state) to 100 (defined as the best imaginable health state) [26].

### 2.6. Adverse Events

Patients were inspected for adverse signs and symptoms such as skin temperature, desaturation, increased heart rate, dyspnea, and fatigue during the treatment or hospitalization time. The severity of these symptoms was assessed and organized into four different categories: (1) Minor and temporary, (2) serious symptoms (potential risk of severe injury or life threatening), (3) manifest injury or disease, and (4) death. All healthcare personnel involved in patient care were tasked with detecting adverse events.

### 2.7. Adherence

Dropouts were registered to evaluate the adherence rate that refers to the total participants who completed the program.

### 2.8. Interventions

All interventions were carried out by the same physiotherapist, once a day, from the second day of hospital admission, and the duration was determined by the length of hospital stay of each patient.

Control Group (CG): The Control Group received the standard medical treatment prescribed by the doctor (consisting of bronchodilators, inhaled corticosteroids and antibiotics) [27].

Global Exercise Group (GEG): All patients received the Control Group treatment added to the global exercise treatment. The program consisted of 15 min of respiratory exercises and 20–30 min of limb exercises. The limb exercises included global active Range of Motion (ROM) exercises, balance and proprioception activities, isometric, flexibility, core stabilization, and aerobic/endurance exercises. The intensity of the treatment was adapted taking into account the subject’s response (the perceived dyspnea and fatigue during the exercise). The intervention was performed following the protocol described by Torres-Sánchez et al. [28].

Functional Electrostimulation Group (FEG): All patients received the Control Group treatment plus Neuromuscular Electrical Stimulation (NEMS) (SEFAR Rehab X2, DJO France S.A.S., Mouguerre, France) superimposed onto voluntary muscular contraction in the quadriceps muscles. The program included 10 min of warm-up (breathing exercises, and 30 min of NMES superimposed onto voluntary muscular contraction). Three different levels of progression were proposed: NMES superimposed onto isometric voluntary muscle contraction with the extended knee; NMES superimposed onto concentric voluntary muscle contraction beginning at 90° of knee flexion; and NMES superimposed onto concentric voluntary muscle contraction of the quadriceps resisted by a low-resistance elastic band. The intervention was performed following the protocol described by Valenza et al. [29].

#### Statistical Analysis

The data obtained were analyzed using Statistical Package for the Social Sciences version 20.0 (International Business Machines, New York, NY, USA). Participant characteristics were determined by descriptive statistics (mean ± standard deviation). Prior to the statistical analysis, the normality of continuous data was assessed by the Kolmogorov–Smirnov test. One-way analysis of variance was used to compare normally distributed baseline demographic variables. Kruskal-Wallis test was used to compare non-normally distributed variables. For each outcome variable measured, a two (preintervention vs. postintervention) × three (treatment groups) repeated measures analysis of variance was performed. Additionally, Bonferroni’s post hoc test was used to identify the specific mean differences if the two-way analysis of variance showed a significant interaction for each variable. The statistical analysis was conducted at 95% confidence level. A *p*-value of less than 0.05 was considered statistically significant.

### 2.9. Sample Size Calculation

The sample size was computed by the lower limb strength. On the basis of previous audit data [30,31], a small positive effect (10 N) was anticipated in the training group. Hence, in order to have 80% power using a two-sided α = 0.05, and a hypothetical drop-out rate of 5%, 12 patients in each group would be needed to show statistically significant differences in lower limb strength between the three groups.

## 3. Results

**Figure 1:**
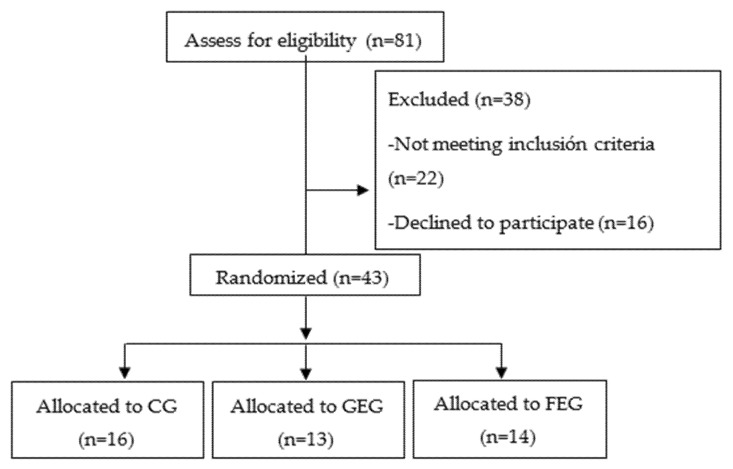
Flow chart.

Of the 81 patients that were assessed for eligibility, a total of 43 patients were randomized in the three groups and performed the intervention with pre- and post-assessment (Figure 1).

The admission characteristics of the subjects for each group are shown in Table 1.

As seen in Table 1, all the included patients were similar in age, BMI, disease severity, quality of life, comorbidities, anxiety and depression, and length of hospitalization, there were no significant differences between groups (*p* > 0.05).

Table 2 shows the mean outcome values at admission among groups.

As we can see in Table 2, there were no significant differences in strength, balance or health related quality of life between groups at admission (*p* > 0.05).

Table 3 shows the main outcomes from admission to discharge in each group.

From admission to discharge, patients in the two experimental groups show significant improvements in all variables, while patients in the control group obtained significant improvements only in daily activities, and anxiety and depression subscales of EQ-5D (*p* < 0.05).

When comparing the groups, there were significant differences in all the variables in favour of the experimental groups (*p* < 0.05). The FEG obtained higher significant values in strength, EQ-5D personal care, and EQ-5D anxiety and depression (*p* < 0.05) compared to GEG. Nevertheless, GEG obtained higher values in balance and EQ-5D VAS compared to the FEG group, but there were no significant differences.

No adverse events were observed during or after the treatment, and all of the patients carried out the program every day during the hospital stay.

## 4. Discussion

The aim of this study was to assess the feasibility and the efficacy of two exercise programs in AECOPD patients with resting hypoxemia. Our results support the feasibility and acceptability of conducting an exercise program in severe AECOPD patients with resting hypoxemia. Moreover, the results of this study showed that there were significant differences between the control group and the two treated groups, in favour of the experimental groups, without adverse events.

Strength, endurance training or physical inactivity can change the properties of the skeletal muscles due to the impressive phenotype plasticity that it possesses. Such adaptations are focused on protecting the functional integrity of the excitation and contraction processes and offering adequate energy supply [32]. Hypoxia may generate several adaptations in the exercising muscle as a progressive increase in ventilation, adaptations of the haematopoietic and cardio-circulatory systems to enhance oxygen delivery to the tissues, and by alterations on the tissue level to optimise oxygen consumption [33,34,35].

Epinephrine levels are increased and closely related to increased lactate levels during submaximal exercise in hypoxia [36]. With adaptation, glycolysis and blood lactate concentrations are reduced, and beta receptors are downregulated [37]. Hypoxic training has been implemented in healthy elderly persons as well as patients with various chronic diseases as coronary artery disease, being scarce in COPD. Burstcher et al. [38] concluded that exercise tolerance increases in patients with COPD after 3 weeks of intermittent hypoxia training, without adverse events.

On the other hand, there is solid evidence of the clinical efficacy of exercise rehabilitation during hospitalization in AECOPD patients [28,38,39,40]. Troosters et al. [41] concluded that resistance global training is an efficacy and safe strategy to decrease the loss of musculoskeletal function during AECOPD. Resistance global training may facilitate functional recovery and generate a protective stimulus to the muscle after an acute exacerbation. In the same way, our results have shown that a global exercise treatment, not only improves the lower limb strength, but also the balance. In older patients with COPD, balance is important in the preservation of functional independence and mobility [42], and is important in the prevention of falls. Hypoxemia generates an impairment on balance greater than that of reduced pulmonary function [43]. Falls are common among people with hypoxic COPD and may lead to vertebral, hip or other fractures with its associated morbidity and mortality risk [44].

In addition, our results have shown that a NEMS program obtained significant betterments in lower limb strength in these patients. A recent Cochrane review has concluded that the use of NMES either alone, or together with conventional exercise training, improve the condition of the peripheral muscles, increase exercise capacity and functional performance, reduce symptoms, and improve health-related quality of life in COPD patients [45]. NMES is an appropriate option that allows the patients to carry out efficient training without intolerable dyspnea.

Additionally, patients in both experimental groups obtained significant betterment in anxiety and depression at discharge compared to the control group. Our results are in line with Gordon et al. [46] that after carrying out a systematic review and meta-analysis, concluded that pulmonary rehabilitation confers significant clinically relevant benefits on anxiety and depression symptoms in COPD patients.

### Study Limitations

Some limitations of this study have to be mentioned, such as the lack of a long-term follow up and the sample size. Additionally, there is subjectivity in the measurement of force with a dynamometer due to the counter resistance of the therapist. Nevertheless, dynamometry has been used to measure the force in COPD patients previously [47]. It is interesting to note that there have been no drop outs in any of the three included groups during the study, a reason could be due to the hospitalization period where the interventions have been developed. Additionally, no adverse events were reported by any patient during the intervention program. Further studies that include COPD patients with resting hypoxemia are needed.

## 5. Conclusions

In conclusion, our results support the feasibility and acceptability of conducting an exercise program in severe AECOPD patients with resting hypoxemia. Additionally, both interventions have improved the lower limb strength, balance, and health related quality of life of these patients. However, comparing both interventions, functional electrostimulation has proven to be more effective in terms of improving health related quality of life and strength than global exercise.

## Figures and Tables

**Table 1:** Admission characteristics of the subjects for each group.

Variables	CG (*n* = 15)	GEG (*n* = 13)	FEG (*n* = 14)	F
Age (years)	70.98 ± 9.22	74.92 ± 7.07	75.80 ± 8.61	12.541
Males (%)	93.3	92.3	85.7	-
BMI (Kg/m^2^)	26.29 ± 3.69	25.41 ± 4.88	23.65 ± 6.25	6.954
FEV1%	33.41 ± 14.97	32.46 ± 12.31	37.12 ± 18.52	12.068
FVC%	41.25 ± 15.36	40.68 ± 12.55	43.58 ± 16.78	11.745
SGRQ	66.79 ± 13.58	63.80 ± 13.57	57.24 ± 11.61	2.582
CCI	4.77 ± 1.40	5.69 ± 1.65	2.80 ± 1.47	14.745
HAD anxiety	7.64 ± 5.17	7.46 ± 5.13	9.31 ± 4.90	2.587
HAD depression	8.13 ± 4.60	7.50 ± 4.70	7.00 ± 4.79	6.135
HAD total	15.92 ± 7.94	14.96 ± 8.60	16.31 ± 8.50	4.203
Hospital stay (days)	11.00 ± 5.29	10.33 ± 3.05	7.47 ± 3.50	8.745

CG: Control Group; GEG: Global Exercise Group; FEG: Functional Electrostimulation Group; BMI: Body Mass Index; FEV1%: Forced Expiratory Volume in 1 s predicted; FVC%: Forced Volumen Capacity Predicted; SGRQ: Saint George Respiratory Questionnaire; CCI: Charlson Commorbidity Index; HAD: Hospital Anxiety and Depression Scale. Data are expressed as mean ± standard deviation.

**Table 2:** Main outcomes at admission in each group.

Variables	CG (*n* = 15)	GEG (*n* = 13)	FEG (*n* = 14)	F
Quadriceps strength (*n*)	107.10 ± 38.03	102.91 ± 40.00	103.93 ± 13.28	1.197
Balance (s)	2.98 ± 4.72	2.15 ± 2.26	2.95 ± 1.99	3.685
EQ-5D mobility	1.84 ± 0.75	1.85 ± 0.49	1.59 ± 0.57	1.679
EQ-5D personal care	1.95 ± 0.58	1.62 ± 0.69	1.89 ± 0.29	2.796
EQ-5D daily activities	2.29 ± 0.68	1.91 ± 0.87	2.12 ± 0.63	2.158
EQ-5D A/D	1.82 ± 0.58	1.51 ± 0.68	1.37 ± 0.49	3.678
EQ-5D pain	2.07 ± 0.67	1.81 ± 0.67	2.18 ± 0.71	2.378
EQ-5D VAS	41.67 ± 15.23	51.23 ± 10.32	44.27 ± 10.23	5.247

CG: Control Group; GEG: Global Exercise Group; FEG: Functional Electrostimulation Group; EQ-5D: EuroQol-5D; A/D: Anxiety and Depression; VAS: Visual Analogue Scale. Data are expressed as mean ± standard deviation.

**Table 3:** Main outcomes from admission to discharge in each group.

Variables	CG (*n* = 15)		GEG (*n* = 13)		FEG (*n* = 14)		F
	Admission	Discharge	Admission	Discharge	Admission	Discharge	
Quadriceps strength (*n*)	107.10 ± 38.03	108.94 ± 69.95	102.91 ± 40.00	118.50 ± 31.64 **	103.93 ± 13.28	133.28 ± 30.41 **	11.046a,b,c
Balance (s)	2.98 ± 4.72	3.12 ± 3.83	2.15 ± 2.26	4.84 ± 7.25 **	2.95 ± 1.99	4.11 ± 2.71 **	3.548a,b
EQ-5D mobility	1.84 ± 0.75	1.51 ± 0.63	1.85 ± 0.49	1.04 ± 0.30 *	1.59 ± 0.57	1.20 ± 0.62 *	0.98ab
EQ-5D personal care	1.95 ± 0.58	1.54 ± 0.82	1.62 ± 0.69	1.39 ± 0.71	1.89 ± 0.29	1.10 ± 0.59 *	1.14abc
EQ-5D daily activities	2.29 ± 0.68	1.91 ± 0.79 *	1.91 ± 0.87	1.19 ± 0.31 *	2.12 ± 0.63	1.08 ± 0.88 *	1.87
EQ-5D A/D	1.82 ± 0.58	1.34 ± 0.39 *	1.51 ± 0.68	1.79 ± 0.80	1.37 ± 0.49	1.29 ± 0.47	2.94b,c
EQ-5D pain	2.07 ± 0.67	2.10 ± 0.81	1.81 ± 0.67	0.91 ± 0.34 *	2.18 ± 0.71	0.79 ± 0.62 **	0.86b
EQ-5D VAS	41.67 ± 15.23	54.14 ± 20.49	51.23 ± 10.32	79.97 ± 14.22 *	44.27 ± 10.23	73.57 ± 13.25 *	19.25ab

CG: Control Group; GEG: Global Exercise Group; FEG: Functional Electrostimulation Group; EQ-5D: EuroQol-5D; A/D: Anxiety and Depression; VAS: Visual Analogue Scale. Data are expressed as mean ± standard deviation. * *p* < 0.05; ** *p* < 0.001. a. Significant differences between CG and GEG. b. Significant differences between CG and FEG. c. Significant differences between GEG and FEG.
